# Supplementary material for: A Likelihood Approach for Real-Time Calibration of Stochastic Compartmental Epidemic Models
Source: PLoS Comput Biol. 2017 Jan 17;13(1):e1005257. doi: 10.1371/journal.pcbi.1005257 (PMC5240920; doi:10.1371/journal.pcbi.1005257)
Supplement: S1 File — (TAR.GZ) [file pcbi.1005257.s014.tar.gz › HSPH_Online-SI-Revision/MSS10/n1-extreme/n1-extreme_NEW_table-4.pdf]

1	R0	{2.95019, 14.2854, 0.1}	{2.41544, 11.8625, 0.12}	{{1.74592, 5.83416, 0.}, {2.96947, 15.963, 0.}}	{18.3059, 27.9481, 200.}	inf	56.	38.5879	{17.5246, 58.5552}	35.479	0.235559	newly	31.	37.91	{16., 67.}	49.3871	0.196402	Reff	2.70237	2.25099	{1.67021, 2.70727}	17.0986	ar	924.	861.6	{743., 940.}	7.5
2	R0	{2.67565, 16.946, 0.1}	{2.49134, 15.6809, 0.04}	{{2.02235, 10.6804, 0.}, {2.93978, 19.9817, 0.}}	{12.0718, 16.8615, 132.}	inf	194.	176.774	{142.684, 218.425}	14.9611	0.0743125	newly	79.	89.36	{74., 104.}	16.5823	0.0657591	Reff	1.69101	1.60845	{1.43104, 1.83647}	8.57286	ar	926.	879.99	{827., 936.}	5.52592
3	R0	{2.40066, 16.6543, 0.1}	{2.06186, 13.5974, 1.01}	{{1.67732, 9.44073, 0.}, {2.76795, 19.6041, 2.}}	{19.032, 26.1031, 976.}	inf	86.	65.0691	{30.864, 105.063}	35.2008	0.212438	newly	43.	39.49	{23., 54.}	23.2326	0.12012	Reff	2.02856	1.77543	{1.48694, 2.25933}	16.5629	ar	916.	778.21	{627., 921.}	15.4618
4	R0	{1.9294, 15.4361, 0.1}	{2.05794, 14.0539, 0.8}	{{1.40191, 4.00571, 0.}, {2.46533, 18.9032, 1.}}	{20.4208, 27.8781, 752.}	inf	96.	62.3127	{22.1224, 93.3153}	36.3737	0.262579	newly	51.	38.44	{22., 53.}	30.8235	0.173028	Reff	1.6207	1.7875	{1.26723, 2.11731}	20.0714	ar	796.	763.36	{501., 904.}	15.9422
5	R0	{2.92859, 19.5393, 0.1}	{1.60401, 6.99905, 0.94}	{{1.44485, 6.00596, 0.}, {1.67405, 7.29751, 1.}}	{45.2291, 64.1796, 860.}	inf	172.	66.7198	{46.418, 88.0736}	61.2094	0.435894	newly	68.	73.25	{49., 95.}	21.9559	0.0991832	Reff	1.94751	1.23424	{1.17642, 1.30224}	36.625	ar	927.	629.55	{551., 710.}	32.0874
6	R0	{1.79622, 12.3148, 0.1}	{1.2165, 3.19514, 0.12}	{{1.06738, 2.19254, 0.}, {1.28596, 3.98513, 1.}}	{32.3089, 74.2662, 198.}	inf	52.	19.4328	{5.32515, 34.0632}	63.9684	0.52964	newly	27.	38.94	{10., 67.}	74.0741	0.277186	Reff	1.38489	0.976955	{0.868826, 1.0251}	29.4559	ar	734.	314.63	{198., 426.}	57.2711
7	R0	{2.45855, 17.1776, 1.}	{1.96022, 9.90573, 1.08}	{{1.25474, 3.26445, 0.}, {2.86656, 18.9813, 2.}}	{28.5966, 45.9381, 98.}	inf	118.	89.1727	{18.2774, 173.393}	48.9402	0.350065	newly	59.	73.52	{40., 104.}	40.6441	0.164545	Reff	1.87833	1.51592	{1.06928, 2.04532}	23.5779	ar	871.	668.65	{348., 934.}	26.6992
8	R0	{2.20009, 15.519, 2.}	{1.75856, 13.6078, 1.75}	{{1.75163, 12.8385, 1.}, {1.81743, 14.0664, 2.}}	{20.0688, 12.3521, 15.5}	inf	146.	90.0422	{68.2808, 110.891}	38.3273	0.218852	newly	70.	49.13	{36., 61.}	30.3857	0.165198	Reff	1.44546	1.25258	{1.21344, 1.29626}	13.3439	ar	839.	706.14	{631., 766.}	15.8355
9	R0	{2.06493, 9.6414, 1.}	{1.3229, 4.27384, 0.4}	{{1.06738, 2.19254, 0.}, {1.86094, 12.272, 2.}}	{37.4995, 69.3449, 106.}	inf	38.	18.5412	{2.25765, 37.7874}	61.8711	0.513442	newly	41.	38.49	{9., 80.}	54.9512	0.310437	Reff	1.89148	1.23352	{1.00195, 1.70445}	35.9295	ar	777.	333.01	{64., 744.}	58.7272
10	R0	{2.60775, 19.6936, 1.}	{1.98222, 16.078, 0.01}	{{1.79436, 13.7237, 0.}, {2.00049, 16.2766, 0.}}	{23.9873, 18.3593, 99.}	inf	154.	106.18	{85.572, 127.888}	31.4208	0.168901	newly	62.	50.98	{37., 63.}	19.4516	0.10026	Reff	1.84629	1.49752	{1.40143, 1.54649}	18.89	ar	889.	792.43	{742., 829.}	10.8628
11	R0	{1.99168, 8.13555, 1.}	{2.31056, 11.8831, 0.56}	{{1.25474, 3.26445, 0.}, {2.98558, 18.1089, 1.}}	{31.9423, 75.4983, 50.}	inf	65.	99.3244	{24.7946, 163.868}	84.2799	0.315718	newly	78.	73.78	{49., 99.}	21.9231	0.121426	Reff	1.67899	1.83716	{1.13805, 2.29036}	23.724	ar	772.	787.37	{462., 943.}	21.7137
12	R0	{1.79304, 9.18941, 0.1}	{2.33599, 14.0168, 0.52}	{{1.79724, 9.44073, 0.}, {2.76795, 18.8445, 1.}}	{31.8859, 55.7775, 532.}	inf	28.	33.5992	{14.3064, 51.288}	43.2854	0.179485	newly	33.	23.18	{10., 35.}	34.9091	0.225122	Reff	1.69442	2.19068	{1.70644, 2.60451}	30.739	ar	725.	845.96	{736., 930.}	18.869
13	R0	{1.84016, 18.3431, 1.}	{1.77899, 16.4604, 1.42}	{{1.42801, 9.02643, 1.}, {1.85141, 18.8228, 2.}}	{3.6911, 12.7745, 58.}	inf	89.	60.4916	{42.9586, 77.9992}	32.2808	0.181333	newly	37.	29.26	{21., 41.}	26.4324	0.13643	Reff	1.48685	1.48734	{1.22157, 1.5637}	4.73939	ar	762.	705.37	{582., 772.}	8.11942
14	R0	{1.94221, 9.85971, 1.}	{2.10885, 14.4588, 1.88}	{{1.94282, 12.1446, 1.}, {2.48947, 17.2836, 2.}}	{10.826, 48.9751, 88.}	inf	111.	117.902	{86.218, 139.917}	17.7759	0.0746665	newly	83.	64.36	{49., 83.}	24.506	0.127576	Reff	1.44306	1.54808	{1.38995, 1.73893}	9.0955	ar	805.	814.11	{751., 890.}	5.5764
15	R0	{1.78578, 12.0541, 0.1}	{1.46344, 9.6891, 0.14}	{{1.38427, 8.26611, 0.}, {1.70497, 15.8627, 0.}}	{19.8745, 31.732, 220.}	inf	101.	42.5645	{21.73, 78.8102}	60.0706	0.441471	newly	70.	32.27	{20., 46.}	53.9	0.367138	Reff	1.39648	1.22619	{1.16232, 1.36352}	14.2273	ar	732.	528.26	{393., 702.}	29.1803
16	R0	{1.94463, 15.6503, 2.}	{2.42923, 12.728, 0.41}	{{1.88486, 8.39294, 0.}, {2.96947, 18.0404, 1.}}	{28.9157, 23.0175, 79.5}	inf	94.	97.1971	{53.8068, 135.01}	27.4153	0.143849	newly	42.	69.54	{49., 94.}	69.	0.228127	Reff	1.60626	1.98713	{1.62382, 2.33101}	26.6655	ar	757.	850.01	{738., 940.}	18.2734
17	R0	{2.08901, 16.4314, 0.1}	{1.98523, 13.9258, 0.06}	{{1.67732, 9.44073, 0.}, {2.24732, 17.1868, 0.}}	{12.4373, 19.9581, 148.}	inf	110.	59.447	{35.1981, 83.0409}	45.9573	0.295373	newly	50.	35.83	{23., 49.}	30.9	0.172248	Reff	1.69628	1.70837	{1.44731, 1.93458}	12.0789	ar	826.	775.89	{669., 868.}	9.5799
18	R0	{2.76419, 18.56, 2.}	{1.96703, 10.5999, 1.}	{{1.24751, 2.5364, 0.}, {2.64334, 18.0404, 2.}}	{29.4529, 43.3876, 52.}	inf	102.	56.4008	{17.6916, 88.5235}	45.9711	0.332132	newly	42.	52.9	{29., 81.}	43.1905	0.148693	Reff	2.30257	1.71796	{1.13607, 2.27342}	26.1597	ar	920.	725.27	{362., 897.}	21.3946
19	R0	{2.3821, 17.1399, 2.}	{1.69258, 7.60934, 0.78}	{{1.22544, 3.07157, 0.}, {2.76849, 17.202, 3.}}	{34.4606, 57.9459, 72.}	inf	70.	42.3965	{10.8824, 93.9447}	58.0055	0.427978	newly	39.	49.82	{22., 80.}	49.0769	0.192078	Reff	2.06528	1.49611	{1.12181, 2.29532}	31.7695	ar	883.	569.43	{275., 915.}	37.1676
20	R0	{2.78314, 19.6127, 0.1}	{2.1432, 16.3137, 0.68}	{{2.01705, 13.609, 0.}, {2.24242, 17.7758, 1.}}	{22.9934, 16.8205, 646.}	inf	270.	164.623	{141.191, 187.85}	39.0286	0.228458	newly	81.	74.52	{60., 90.}	13.9012	0.0655169	Reff	1.35817	1.2664	{1.16156, 1.32152}	6.75707	ar	943.	821.28	{781., 870.}	12.9077
21	R0	{1.85124, 14.6794, 0.1}	{1.62521, 12.2636, 1.6}	{{1.22544, 2.5364, 0.}, {2.036, 18.765, 2.}}	{17.602, 34.3062, 1548.}	inf	54.	39.1319	{10.2777, 66.7501}	40.3653	0.286525	newly	27.	26.81	{14., 42.}	36.5556	0.16827	Reff	1.61058	1.43226	{1.11444, 1.7558}	15.7205	ar	730.	595.16	{247., 805.}	23.7151
22	R0	{2.1028, 14.9852, 2.}	{2.13986, 12.3419, 0.81}	{{1.77826, 9.06807, 0.}, {2.64334, 17.2462, 1.}}	{12.7552, 24.1502, 59.5}	inf	146.	133.186	{73.3918, 195.177}	27.7616	0.139447	newly	80.	83.97	{60., 106.}	18.1625	0.0778846	Reff	1.46565	1.50507	{1.33562, 1.72939}	7.9141	ar	840.	810.21	{695., 908.}	8.51548
23	R0	{1.86601, 10.2157, 1.}	{2.2762, 13.106, 0.82}	{{1.77826, 8.56054, 0.}, {2.93488, 18.8445, 1.}}	{26.0993, 38.6312, 38.}	inf	53.	71.3553	{38.3391, 104.312}	48.2397	0.17651	newly	50.	48.87	{34., 64.}	19.46	0.0892415	Reff	1.64022	1.94921	{1.57638, 2.42148}	22.5595	ar	747.	823.16	{721., 931.}	15.6037
24	R0	{2.34032, 10.0992, 0.1}	{2.36666, 10.891, 0.16}	{{1.737, 5.43578, 0.}, {2.86001, 15.5255, 1.}}	{13.2592, 30.4101, 228.}	inf	45.	56.5389	{26.6244, 81.8975}	42.2984	0.165854	newly	41.	56.12	{29., 81.}	50.1463	0.168924	Reff	2.12969	2.12123	{1.5981, 2.52767}	11.6662	ar	887.	846.63	{699., 927.}	7.42841
25	R0	{1.91548, 15.3401, 1.}	{2.07394, 13.745, 0.17}	{{1.71608, 9.75208, 0.}, {2.30108, 17.1709, 1.}}	{12.6072, 15.7478, 83.}	inf	62.	62.5002	{43.6074, 78.341}	21.5682	0.0948243	newly	31.	39.06	{25., 52.}	33.7419	0.122969	Reff	1.66455	1.79806	{1.56091, 2.01472}	11.5262	ar	765.	796.96	{703., 874.}	7.9085
26	R0	{2.75283, 10.6721, 0.1}	{2.69877, 16.7832, 0.07}	{{2.11132, 12.644, 0.}, {2.96978, 18.8857, 0.}}	{9.85227, 57.8453, 156.}	inf	42.	61.4935	{38.7404, 83.2089}	49.9657	0.167764	newly	48.	34.77	{23., 46.}	30.3125	0.166421	Reff	2.52985	2.42442	{1.94083, 2.67243}	8.6163	ar	926.	905.8	{832., 947.}	3.67171
27	R0	{2.08367, 19.7849, 1.}	{1.55781, 9.62104, 1.2}	{{1.22544, 3.07157, 0.}, {1.93882, 15.8893, 2.}}	{25.2372, 51.3718, 102.}	inf	122.	66.8098	{9.20088, 130.036}	47.7696	0.445173	newly	56.	48.13	{21., 72.}	32.2679	0.188554	Reff	1.4669	1.16822	{0.963401, 1.37808}	20.3614	ar	833.	551.37	{216., 792.}	33.8187
28	R0	{2.64334, 17.1765, 0.1}	{1.66974, 8.3766, 0.93}	{{1.25474, 3.26445, 0.}, {2.21411, 14.4607, 2.}}	{37.3957, 52.4985, 936.}	inf	150.	64.7614	{13.7243, 133.439}	58.6936	0.506907	newly	88.	57.05	{25., 82.}	35.9432	0.232717	Reff	1.90056	1.32327	{1.03978, 1.66867}	30.5974	ar	934.	601.94	{339., 860.}	35.591
29	R0	{2.87008, 13.3872, 0.1}	{2.24313, 18.5194, 0.02}	{{2.23915, 18.4504, 0.}, {2.27582, 19.0665, 0.}}	{21.8445, 38.3365, 116.}	inf	74.	79.2061	{65.6325, 92.8115}	14.5582	0.059417	newly	61.	35.48	{24., 45.}	41.8361	0.246285	Reff	2.46827	1.91708	{1.86543, 1.97493}	22.3308	ar	941.	852.39	{824., 882.}	9.41658
30	R0	{2.06105, 19.6275, 1.}	{1.52597, 8.77873, 0.92}	{{1.40191, 6.71734, 0.}, {1.54207, 9.08517, 1.}}	{25.9616, 55.2733, 16.}	inf	138.	70.1728	{50.6088, 90.8415}	49.1502	0.31243	newly	44.	52.42	{38., 68.}	26.5	0.101492	Reff	1.05732	0.88531	{0.858403, 0.905507}	16.3123	ar	747.	601.95	{506., 664.}	19.4257
31	R0	{2.30764, 13.435, 2.}	{2.03859, 11.7028, 1.18}	{{1.21159, 2.5364, 0.}, {2.86095, 19.6025, 2.}}	{26.3072, 46.1085, 47.}	inf	103.	74.6419	{13.3665, 130.232}	43.7072	0.343591	newly	60.	54.35	{26., 81.}	29.95	0.166915	Reff	1.88303	1.69183	{1.08762, 2.23342}	22.7673	ar	877.	691.48	{308., 930.}	25.0103
32	R0	{2.30565, 11.9237, 0.1}	{1.79392, 9.67089, 1.11}	{{1.28596, 3.98513, 0.}, {2.29887, 16.6365, 3.}}	{25.8751, 40.6089, 1084.}	inf	62.	55.2639	{15.7662, 98.8689}	47.9154	0.282185	newly	41.	47.19	{24., 68.}	34.7561	0.15618	Reff	1.97825	1.53676	{1.16698, 1.91848}	24.3379	ar	853.	645.52	{365., 875.}	25.8148
33	R0	{2.24031, 18.582, 1.}	{1.91788, 16.5561, 0.15}	{{1.75668, 13.8428, 0.}, {2.2194, 19.1478, 1.}}	{14.392, 12.3475, 85.}	inf	83.	67.1323	{49.5733, 97.1059}	26.4977	0.135835	newly	35.	32.51	{20., 43.}	22.3714	0.10995	Reff	1.90874	1.66228	{1.54515, 1.8437}	12.9125	ar	864.	781.12	{731., 844.}	9.7662
34	R0	{2.33306, 15.9759, 0.1}	{1.92196, 11.9344, 1.25}	{{1.22544, 3.07157, 0.}, {2.48947, 19.255, 2.}}	{20.8053, 37.4513, 1212.}	inf	138.	68.3648	{13.2914, 104.665}	50.5548	0.413083	newly	70.	47.84	{30., 64.}	38.2857	0.227054	Reff	1.80112	1.60083	{1.10231, 2.01519}	18.1159	ar	854.	679.9	{263., 903.}	23.2436
35	R0	{2.48357, 18.3573, 1.}	{2.12867, 11.6918, 1.06}	{{1.24751, 2.5364, 0.}, {2.86095, 19.9929, 2.}}	{25.3456, 40.5174, 88.}	inf	114.	88.1201	{16.792, 155.132}	44.3716	0.339804	newly	58.	65.75	{46., 82.}	25.9138	0.104446	Reff	1.98686	1.72253	{1.11273, 2.21173}	20.9074	ar	835.	725.58	{362., 942.}	23.1066
36	R0	{2.59548, 19.9046, 0.1}	{1.83598, 11.2395, 0.12}	{{1.67732, 9.44073, 0.}, {2.04669, 15.0173, 1.}}	{29.2623, 43.5332, 198.}	inf	110.	75.5434	{44.5949, 98.7037}	34.0357	0.192071	newly	53.	54.29	{40., 69.}	17.2642	0.0756722	Reff	2.02967	1.4952	{1.37965, 1.65161}	26.3329	ar	863.	731.92	{652., 833.}	15.5041
37	R0	{2.29641, 16.9868, 0.1}	{1.55816, 9.20342, 0.05}	{{1.22544, 3.07157, 0.}, {1.8951, 15.9062, 0.}}	{32.1478, 45.8202, 142.}	inf	149.	74.47	{16.352, 145.536}	50.0974	0.444328	newly	59.	61.05	{29., 84.}	30.5932	0.145454	Reff	1.61208	1.19709	{1.00818, 1.36489}	25.7426	ar	877.	584.6	{333., 798.}	33.3409
38	R0	{2.65743, 15.4569, 1.}	{2.36102, 16.577, 0.55}	{{2.11292, 12.9719, 0.}, {2.56757, 18.9032, 1.}}	{12.004, 17.3108, 45.}	inf	119.	143.67	{103.178, 179.642}	26.2632	0.0994971	newly	62.	68.69	{53., 84.}	18.8871	0.0758117	Reff	1.96118	1.67899	{1.5369, 1.78865}	14.3888	ar	897.	866.3	{796., 921.}	4.58194
39	R0	{2.67953, 14.5132, 2.}	{2.56034, 15.6401, 0.09}	{{1.93539, 9.63533, 0.}, {2.96978, 18.8857, 0.}}	{12.4326, 22.1969, 95.5}	inf	36.	57.0151	{30.1847, 81.5155}	66.0697	0.210055	newly	28.	34.25	{18., 52.}	36.6071	0.137314	Reff	2.47857	2.30937	{1.8254, 2.65523}	11.4017	ar	894.	886.53	{787., 946.}	5.3434
40	R0	{1.94594, 11.928, 1.}	{1.88822, 9.44571, 0.78}	{{1.25474, 3.26445, 0.}, {2.86095, 18.0404, 2.}}	{30.3875, 52.1201, 76.}	inf	81.	121.103	{22.6932, 254.099}	101.327	0.380436	newly	45.	84.59	{44., 113.}	96.7333	0.301692	Reff	1.35827	1.18685	{0.923757, 1.47922}	17.2749	ar	796.	657.08	{381., 921.}	28.3467
41	R0	{1.66297, 10.0047, 0.1}	{1.6416, 19.0351, 3.88}	{{1.64587, 19.5316, 4.}, {1.66638, 19.7105, 4.}}	{1.44099, 90.2619, 3780.}	inf	49.	75.5637	{56.4645, 96.6021}	54.7212	0.181036	newly	37.	29.17	{20., 39.}	23.9189	0.127396	Reff	1.32539	1.26431	{1.23737, 1.28973}	4.60843	ar	716.	656.31	{591., 710.}	8.75
42	R0	{2.91568, 16.4453, 1.}	{2.5005, 15.7189, 1.8}	{{2.17882, 12.0512, 1.}, {2.86095, 18.9813, 2.}}	{14.3934, 15.082, 94.}	inf	83.	81.8466	{51.3238, 113.03}	23.9506	0.116669	newly	52.	46.77	{35., 60.}	20.5962	0.0996253	Reff	2.50748	2.14664	{1.92696, 2.40691}	14.4063	ar	919.	875.55	{837., 937.}	5.54407
43	R0	{2.55898, 15.8889, 0.1}	{2.56164, 15.1267, 0.07}	{{1.93539, 9.63533, 0.}, {2.96797, 18.4042, 0.}}	{13.5108, 18.7273, 156.}	inf	88.	61.3742	{34.4501, 87.7386}	32.4596	0.192581	newly	64.	38.23	{25., 52.}	40.8906	0.245489	Reff	2.22119	2.28684	{1.79451, 2.64689}	13.2367	ar	904.	887.69	{790., 946.}	5.33739
44	R0	{2.28649, 15.9017, 0.1}	{2.65869, 16.7025, 0.02}	{{2.29055, 13.7413, 0.}, {2.96978, 18.8857, 0.}}	{17.6615, 15.1849, 116.}	inf	132.	152.213	{98.1972, 204.676}	28.6735	0.11336	newly	68.	74.88	{58., 96.}	17.3824	0.0695093	Reff	1.71487	1.92951	{1.75555, 2.07312}	13.4608	ar	880.	901.04	{841., 945.}	4.91364
45	R0	{2.65121, 18.6339, 0.1}	{2.32868, 12.1305, 0.42}	{{1.80312, 6.16453, 0.}, {2.96947, 17.2462, 1.}}	{16.3166, 35.0954, 442.}	inf	87.	52.8719	{20.682, 79.2939}	39.8232	0.274661	newly	50.	42.65	{21., 62.}	30.54	0.168323	Reff	2.3092	2.10057	{1.66679, 2.59971}	14.1597	ar	928.	840.66	{749., 934.}	9.79957
46	R0	{2.52291, 18.0251, 2.}	{1.71705, 7.25311, 0.13}	{{1.25474, 3.26445, 0.}, {2.46477, 18.9032, 1.}}	{33.1456, 61.2565, 93.5}	inf	129.	50.8117	{8.43456, 115.629}	61.5676	0.542919	newly	55.	64.37	{24., 109.}	44.7818	0.195673	Reff	1.99814	1.47914	{1.12412, 2.02412}	27.353	ar	894.	610.87	{325., 893.}	32.0951
47	R0	{2.22618, 17.1225, 0.1}	{1.99171, 12.3096, 0.62}	{{1.44485, 5.4396, 0.}, {2.76795, 19.6041, 2.}}	{19.4515, 33.4538, 620.}	inf	75.	55.057	{24.5232, 93.6097}	38.4428	0.247436	newly	39.	38.54	{23., 52.}	26.6154	0.123847	Reff	1.91451	1.74196	{1.29777, 2.29693}	17.0278	ar	850.	743.76	{532., 912.}	15.9788
48	R0	{2.04405, 16.073, 1.}	{2.13523, 16.621, 3.}	{{1.77553, 12.6465, 3.}, {2.39393, 19.7817, 4.}}	{9.29187, 12.5063, 206.}	inf	64.	100.29	{81.8242, 118.069}	59.51	0.200775	newly	41.	48.82	{37., 61.}	23.9024	0.0908385	Reff	1.69452	1.68947	{1.46412, 1.89215}	6.03487	ar	779.	816.85	{740., 885.}	8.82285
49	R0	{2.84396, 18.097, 1.}	{1.26309, 3.38932, 0.21}	{{1.06738, 2.19254, 0.}, {1.47488, 4.00571, 1.}}	{55.5869, 81.2714, 97.}	inf	134.	25.6922	{3.39704, 49.4196}	80.8267	0.906848	newly	69.	55.33	{10., 103.}	47.8986	0.328668	Reff	2.18132	1.10021	{0.932228, 1.2821}	49.5622	ar	952.	336.64	{115., 526.}	64.6387
50	R0	{1.85341, 18.0975, 4.}	{1.24336, 3.27479, 0.07}	{{1.21159, 2.5364, 0.}, {1.28596, 3.98513, 0.}}	{32.9151, 81.9048, 98.25}	inf	138.	26.7943	{8.72733, 43.5342}	80.5839	0.786224	newly	56.	53.44	{17., 92.}	40.5	0.230735	Reff	1.27329	0.992408	{0.954162, 1.03266}	22.0597	ar	754.	353.91	{213., 471.}	53.0623
